# MLKL regulates Cx43 ubiquitinational degradation and mediates neuronal necroptosis in ipsilateral thalamus after focal cortical infarction

**DOI:** 10.1186/s13041-023-01064-4

**Published:** 2023-10-30

**Authors:** Yanyan Tang, Quanhong Chu, Guanfeng Xie, Yafu Tan, Ziming Ye, Chao Qin

**Affiliations:** Department of Neurology, First Affiliated Hospital of Guangxi Medical University, 22 Shuangyong Road, Nanning, Guangxi Province 530021 China

**Keywords:** Cerebral infarction, Secondary damage, Necroptosis, MLKL, Cx43

## Abstract

Necroptosis is known to play an important role in the pathophysiology of cerebral ischemia; however, its role in the occurrence of secondary thalamic injury after focal cerebral infarction and the mechanism about how mixed lineage kinase domain-like (MLKL) executes necroptosis in this pathophysiology are still unclear. In this study, Sprague-Dawley rats were subjected to distal branch of middle cerebral artery occlusion (dMCAO). The expression of MLKL, connexin 43 (Cx43) and Von Hippel-Lindau (VHL) in vitro and in vivo were assessed by Western blot. Bioinformatic methods were used to predict the potential binding sites where MLKL interacted with Cx43, and the ubiquitination degradation of Cx43 regulated by VHL. The interactions among MLKL, Cx43, VHL, and Ubiquitin were assessed by immunoprecipitation. Dye uptake assay were used to examine the Cx43 hemichannels. Intracellular Ca^2+^ concentration was measured using Fluo-4 AM. Overexpression and site-directed mutagenesis studies were used to study the mechanisms by which MLKL regulates Cx43 ubiquitinational degradation to mediate neuronal necroptosis. We found that MLKL and Cx43 were upregulated in the ventral posterolateral nucleus (VPN) of the ipsilateral thalamus after dMCAO. In the in vitro experiments MLKL and Cx43 were upregulated after TSZ-mediated necroptosis in SH-SY5Y cells. The interaction between MLKL and Cx43 inhibited the K48-linked ubiquitination of Cx43 in necroptotic SH-SY5Y cells. VHL is an E3 ubiquitin ligase for Cx43, and MLKL competes with VHL for binding to Cx43. Interaction of MLKL Ser454 with Cx43 can trigger the opening of Cx43 hemichannels, causing increased intracellular Ca^2+^, and cell necroptosis. This innovative study at animal models, cellular, and molecular levels is anticipated to clarify the roles of MLKL and Cx43 in thalamic damage after focal cortical infarction. Our findings may help identify novel targets for neurological recovery after cortical infarction.

## Introduction

Cerebral infarction is a major cause of mortality and disability in adults. Despite considerable advances in acute vascular recanalization, approximately 50% of survivors develop permanent disability, imposing a heavy burden on the affected individuals, families, and the society at large [1]. Therefore, development of appropriate interventions to reverse neurological deficit and improve the quality of life of patients with cerebral infarction is a key imperative.

Available evidence suggests that cortical cerebral infarction leads to neuronal damage not only at the primary lesion site, but also in remote non-ischemic regions [2–6]. This phenomenon, also referred to as secondary degeneration or secondary damage, has been found to be involved in post-stroke cognitive impairment and vascular Parkinson’s syndrome [3, 7–9]. Secondary degeneration is one of the important mechanisms that hamper functional recovery after cerebral infarction [10–13]. We and many other groups have previously demonstrated secondary damage of the ipsilateral ventroposterior nucleus (VPN) in the thalamus after acute ischemic stroke [14–19]. Owing to its delayed onset, secondary degeneration is a potential target for extending the therapeutic window of acute ischemic stroke.

A range of mechanisms have been implicated in the causation of secondary damage, including apoptosis [19, 20], retrograde degeneration [3, 8, 16] autophagy [18], inflammation [21], oxidative stress [22], excitotoxicity [17], and others [4]. Necroptosis is a lytic form of caspase-independent programmed cell death, and misactivation of necroptosis has been found to be involved in many pathological conditions. Programmed necroptosis induced by the tumor necrosis factor alpha (TNF-α) cytokine family relies on a kinase cascade consisting of receptor interacting protein-1 (RIP1) and receptor interacting protein-3 (RIP3). RIP3 promotes the phosphorylation of mixed lineage kinase domain-like protein (MLKL). Phosphorylated MLKL forms an oligomer that binds to phosphatidylinositol lipids and cardiolipin, which disrupts the integrity of cell membrane, ultimately leading to necroptosis [23–25]. Recent studies have shown increased expression of RIP1, RIP3, MLKL, as well as the interaction of RIP1 and RIP3 in rat models of cerebral infarction, indicating the involvement of necroptosis in the process of cerebral ischemia [26, 27]. Necrostatin-1, a specific inhibitor of necroptosis, showed a neuroprotective effect after cerebral ischemia in a rat model of middle cerebral artery occlusion (MCAO) [26, 28]. The above findings indicate an important role of necroptosis in the development of ischemic stroke. However, the role of necroptosis in secondary degeneration after cerebral infarction remains unclear.

MLKL, as a core regulator in necroptosis, acts as an executioner of necroptosis. The oligomerized MLKL proteins translocate to plasma membrane, and form channels or pores, disrupting the membrane integrity and ultimately causing cell necrosis [25, 29–32]. The mechanism by which MLKL works on membranes and causes cell death is not clear. Of note, the membrane localization of MLKL promotes Ca^2+^ influx, which is an early event in necroptosis [33–35]. However, other studies have found that MLKL forms cation channels that are permeable preferentially to Mg^2+^, Na^+^, and K^+^ rather than Ca^2+^ in necroptotic cell death [30, 36]. These conflicting results prompted us to speculate the potential involvement of Ca^2+^-permeable channels downstream of MLKL.

Cx43 is one of the most important connexins on the plasma membrane and involved in the formation of cell gap junctions/non-junctional hemichannels that provide a pathway for the exchange of ions and metabolites between the cytoplasm and extracellular milieu [37]. Under pathological conditions, such as ischemia and hypoxia, a large number of Cx43 hemichannels are opened [38], thereby increasing the intracellular Ca^2+^ concentration [39, 40]. Cx43 has been shown to have a short half-life in cultured cells and in vivo experiments (1.5-5.0 h) [41, 42]. Ubiquitin is known to regulate the functions of Cx43 gap junctions. K63-ubiquitylated on K264 and K303 of Cx43 is critical for the internalization of Cx43 gap junctions [43]. The internalization and ubiquitination degradation of Cx43 may affect intracellular Ca^2+^ concentration. In a lipopolysaccharide-induced acute lung injury model of newborn Sprague-Dawley rats, the expression of necroptosis-related markers (RIP1, RIP3, and MLKL) in the bronchoalveolar lavage fluid was significantly up-regulated after lipopolysaccharide induction, accompanied by up-regulation of Cx43 protein. Moreover, adenoviral overexpression of Cx43 was found to exacerbate necroptosis in lung tissue [44]. Despite these advances, there is no clear evidence of a direct association between Cx43 and MLKL-dependent necroptosis. Therefore, we hypothesized that the expression of MLKL in the ipsilateral thalamus is significantly up-regulated after cerebral infarction, and that MLKL interacts with Cx43 and regulates opening of Cx43 hemichannels, leading to intracellular calcium overload and neuronal necroptosis.

## Materials and methods

### Animals and ethics statement

Adult male SD rats (weight: 280–330 g; age: 10–12 weeks) were provided by the Experimental Animal Center of the Guangxi Medical University (Guangxi, China). The rats were housed in a controlled environment (12-hour light/dark cycle; temperature: 25 ± 2 ℃) with *ad libitum* access to water and food. All animal experiments were approved by the Animal Care & Welfare Committee of the Guangxi Medical University (Project Proposal number 202,106,007) and carried out in accordance with the Guidelines for the Care and Use of Experimental Animals (National Institute of Health, Bethesda, MD, USA).

### Animal model

Permanent occlusion of the distal branch of middle cerebral artery (dMCAO) surgery was carried out by monopolar electrocoagulation, as described elsewhere [3, 8, 15]. Briefly, anesthesia was induced with isoflurane (3–4%) in 100% oxygen (3 L/min) and maintained with isoflurane (1.5–2.5%) in 100% oxygen (800 mL/min), delivered through a nasal mask during surgery. The distal striatal branch of the MCA was exposed under a surgical microscope, followed by its occlusion using monopolar coagulation. Sham-operated animals underwent the same surgical procedure except for dMCA coagulation. After surgery, neurological function of SD rats was evaluated for neurological function and rats with no cortical infarction or neurological deficit were excluded from the subsequent experiments.

### Immunofluorescence staining

Rats were randomly divided into two groups for immunofluorescence experiments: Sham operation group and dMCAO group. The animals were sacrificed at 2 weeks after dMCAO, and intracardiac perfusion was performed with 0.9% saline and 4% paraformaldehyde in phosphate-buffered saline (PBS) (0.01 M, pH 7.4). The brain tissues were quickly removed and fixed in 10, 20, and 30% sucrose in the same fixative for cytoprotection. After fixation, the brains were frozen at − 20 °C and sliced into 30-μm-thick coronal sections with cryotome (Leica, Wetzlar, Hessen, Germany). Sections selected from the typical thalamus [bregma: between anterior-posterior (AP)-2.3 mm to-4.3 mm] were used.

Triple-fluorescent immunohistochemistry was performed to identify the cell types in which MLKL is expressed and to determine the exact location of MLKL expression. The detailed procedure is described elsewhere [15, 35]. NeuN, glial fibrillary acidic protein (GFAP), and CD68 were used as markers of neuronal nuclei, astrocytes, and microglia respectively. The primary antibodies used in these studies included rabbit anti-MLKL (1:300, Thermo, Cat# PA5102810, RRID: AB_2852200), mouse anti-NeuN (1:1000; Millipore, Cat# MAB377, RRID: AB_2298772), mouse anti-CD68 (1:100; Millipore, Cat# MAB1435, RRID: AB_177576) and mouse anti-GFAP (1:1000, Millipore, Cat# AB5804, RRID: AB_11212369). After rinsing in 0.01 M PBS, the sections were incubated for 1 h at room temperature with the following secondary antibodies: Goat anti-Rabbit IgG H&L (Alexa Fluor® 488) (1:100; Abcam, Cat#: ab150077, RRID: AB_2630356), and Goat anti-Mouse IgG, Cy3 conjugate (1:100; Millipore, Cat# AP124C, RRID: AB_92459). After incubation with IgG antibody, sections were washed with PBS and mounted with mounting medium containing 4′,6-diamidino-2-phenylindole (DAPI, Solarbio, Beijing, China, Cat# S2110). Slides were analyzed with a confocal laser microscope (SP8, Leica Microsystems, Wetzlar, Hessen, Germany). The co-localization analysis of MLKL and NeuN, CD68 or GFAP was performed within 6 non-repeated rectangular areas in the ipsilateral VPN after dMCAO. Images were analyzed with ImageJ software.

### Western blot

Proteins of thalamic VPN subregion were extracted and Western blot assay was performed, as previously described [15]. Brain tissues and SH-SY5Y cell were lysed with RIPA buffer (Beyotime, Shanghai, China). Samples were centrifuged and then the supernatant was collected. Protein concentration was determined using bicinchoninic acid (BCA) method, as recommended by the manufacturer’s (Beyotime). Proteins were denatured by boiling for 10 min, then loaded on 8–12% sodium dodecyl sulfate-polyacrylamide gel electrophoresis (SDS-PAGE), and then transferred from the gels to polyvinylidene fluoride (PVDF) membranes (MilliporeSigma, Burlington, MA, USA). The membranes were blocked with 5% skimmed milk or bovine serum albumin, and then incubated overnight with the following primary antibodies at 4 °C: rabbit anti-MLKL (1:300, Thermo, Cat# PA5102810, RRID:AB_2852200), rabbit anti-Phospho-MLKL Antibody (1:1000, Affbiotech, Cat#AF7420, RRID: AB_2843860), rabbit monoclonal anti-Connexin 43/GJA1 antibody (1:1000, Abcam, Cat# ab235585), rabbit anti-GAPDH antibody (1:10000, Abcam, Cat# ab181602, RRID:AB_2630358), rabbit anti-VHL (1:1000, Genetex, Cat# GTX101087), rabbit anti-K63-linkage Specific Polyubiquitin (1:1000, Cell Signaling Technology, Cat# 5621, RRID:AB_10827985), rabbit anti-K48-linkage Specific Polyubiquitin (1:1000, Cell Signaling Technology, Cat# 8081, RRID:AB_10859893), Ubiquitin (P4D1) Mouse mAb (1:1000, Cell Signaling Technology Cat# 3936, RRID:AB_331292), rabbit anti-His-Tag (Cell Signaling Technology Cat# 12,698, RRID:AB_2744546), anti-FLAG-tag (Cell Signaling Technology Cat# 14,793, RRID:AB_2572291), rabbit anti-V5-Tag (Cell Signaling Technology Cat# 13,202, RRID:AB_2687461), mouse anti-HA-Tag (Sigma-Aldrich Cat# H9658, RRID:AB_260092), anti-Myc-Tag (Cell Signaling Technology Cat# 2278, RRID:AB_490778), and anti-GAPDH (Cell Signaling Technology Cat# 5174, RRID:AB_10622025). The blots were washed 3 times with TBST and incubated for 2 h with the following secondary antibodies at room temperature: HRP-conjugated goat anti-rabbit secondary antibody (1:5000, Thermo Fisher Scientific, Cat# C314660) and HRP-conjugated goat anti-mouse secondary antibody (1:5000, Thermo Fisher Scientific, Cat# C3146601). Protein bands were visualized using enhanced chemi-luminescence reaction (ECL) and analyzed by ImageJ software [35].

### Molecular docking analysis

To predict the binding site between Cx43 and MLKL, we performed molecular docking analysis based on the modeling substance structure. First, the three-dimensional structures of the Cx43 carboxy-terminal (ID: 1R5S; organism: Rattus norvegicus; www.rcsb.org/) and MLKL protein (ID: 4BTF; organism: Mus musculus; www.rcsb.org/) were downloaded from the PDB database (file format: PDB). The AutoDock software [45] was used to preprocess the protein three-dimensional structure of Cx43 carboxyl terminus and MLKL. The preprocessing steps included removal of all water molecules and other heteroatoms in the files of Cx43 and MLKL. Then, polarized hydrogen was added and the charge was calculated. MLKL was defined as receptor and Cx43 carboxy terminus as ligand. The maximum number of conformations generated by molecular docking was set to 20 models and the Lamarckian genetic algorithm (LGA) was used for global search. Finally, PyMoL 2.3 software (Schrödinger, Inc., New York, NY, USA) [46] was used for detailed analysis, and the key residues of Cx43 carboxyl terminus and MLKL were determined.

### Cell culture

SH-SY5Y human neuroblastoma cells were purchased from American Type Culture Collection (ATCC, Manassas, VA, USA). The cell line was cultured in DMEM containing 10% FBS and 1% penicillin/streptomycin and incubated at 37 °C and 95% O_2_, 5% CO_2_. Cells were grown to 80% confluence in 25 cm^2^ culture flasks.

### Plasmid transfection

The following plasmids were constructed and purchased from GeneChem (Shanghai, China): plasmids (CMV-MCS-3FLAG-SV40-neomycin) containing the full length of human MLKL coding sequence (gene ID: 197,259) and its mutant form (gene ID: 197,259 (S454A)) or control plasmids; the plasmids (CMV-MCS-Myc-SV40-neomycin) containing the full length of human GJA1 coding sequence (gene ID: 2697) and its mutant form (gene ID: 2697 (K303G)) or control plasmids; the plasmids (CMV-MCS-HA-SV40-neomycin) containing human Ub coding sequence (gene ID: 7316); and the plasmids (CMV-MCS-V5-SV40-neomycin) containing human VHL coding sequence (gene ID: 7428). TurboFect Transfection Reagent (Thermo, Cat# R0531) and plasmids were prepared in the following ratios: plasmid (1 μg): TurboFect Transfection Reagent (3 μL). After 20-minute incubation, the mixture was added to the cells seeded in the dish and left for 48 h for further analysis, with appropriate replacement of fresh medium. The final concentration of plasmid was 0.8 μg/mL.

### Flow cytometry analysis for assessment of cellular necroptosis

Flow cytometry analysis was used to assess necroptosis in cellular experiments. Briefly, SH-SY5Y cells were trypsinized and centrifuged at 1000 rpm for 5 min. The cells were resuspended in 500 μL binding buffer at a concentration of 1 × 10^6^ cells/mL. After 2 washes with PBS, 5 μL of propidium iodide (PI) and 5 μL of AnnexinV-FITC were added to the cells and incubated for 15 min at room temperature in dark. Cells were analyzed by flow cytometry within 1 h, and double-labeled (PI^+^ /Annexin V^+^) cells were considered as necroptotic cells [47].

### Co-immunoprecipitation analysis and detection of ubiquitination

For Co-IP, a mixture of dynabeads (Invitrogen, USA) and antibody was prepared and incubated for 24 h. Then, the extracted cellular proteins were added and the mixture (dynabeads containing specific antibodies and extracted cellular proteins) was incubated on a rotator at 4 °C for 2 h. After incubation, the mixture was washed thrice with PBST. The mixture was then suspended with SDS blue loading buffer followed by a boiling water bath for 5 min. After centrifugation, the Dynabeads were discarded, and the supernatant was used for further Western blot analysis. In the Western blot analysis, the supernatant was used to detect ubiquitination of target protein with anti-Ub antibody. All co-immunoprecipitation experiments were repeated three times.

### Cell viability assay

The inhibitory effect of TSZ [20 ng/mL TNF-ɑ (human), 100 nM Smac mimetics and 20 μM Z-VAD(OH)-FMK] on SH-SY5Y cells was assessed by CCK-8 assay (Cell Counting Kit-8, DOJINDO, Japan) according to the manufacturer’s protocol. Cells (1.5 × 10^3^ cells/mL) were placed in 96-well plates and treated with TSZ for 10 h in the incubator. After intervention in different groups, 100 μL of CCK-8 dilution solution was added to each group, and cells were cultured at 37 °C for 2 h. Absorbance was measured at 450 nm using a Thermo Microplate Reader (Thermo, USA).

### Assay of lactate dehydrogenase release

Lactate dehydrogenase (LDH) release is an indicator of cellular necrosis. Culture medium was collected for analysis of LDH release using a colorimetric assay kit (Jiancheng, Nanjing, China) following the manufacturer’s protocol. Released LDH was measured by a coupled enzymatic reaction, which results in the conversion of the tetrazolium salt to red formazan by diaphorase. Briefly, 120 μL of cell culture medium was mixed with 60 μL of LDH working solution and incubated for 60 min at room temperature in dark. Absorbance was measured at 490 nm by Thermo Microplate Reader (Thermo, USA). The percentage of LDH release was calculated according to the formula provided by the kit supplier.

### Dye uptake assay

SH-SY5Y cells were grown to 85% confluence and then subjected to TSZ for 10 h. The cells were then incubated with 5 μM ethidium bromide (EB) in HBSS (without Ca^2+^) for 10 min. EB was used as a tracer for hemichannel activity. The cells were then rinsed thrice with HBSS (with Ca^2+^), respectively, and subjected to fluorescence imaging analysis with Leica SP8 confocal microscope (Wetzlar, Hessen, Germany). The excitation light was 488 nm, and the emission light was 555 nm. Images obtained from 6 randomly selected non-overlapping fields of culture dish were analyzed by ImageJ software [35].

### Determination of cytoplasmic Ca^2+^ concentration in SH-SY5Y cells

For the assessment of cytosolic Ca^2+^ changes in SH-SY5Y cells, Fluo-4AM kit (Thermo, USA, Cat# F142) was used following the manufacturer’s instruction. After rinsing the cells thrice with HBSS, the cells were loaded for 35 min with the acetoxymethyl ester forms of Fluo-4 working solution [4.5 μL Pluronic F-127 (10%) + 10 μL Fluo-4AM (3mM) + 3.575 mL HBSS] at 37 °C in the incubator, followed by a 30 min de-esterification period. The fluorescence signal intensity was measured with Leica SP8 confocal microscope (Wetzlar, Hessen, Germany). The excitation light was 488 nm and the emission light was 520–530 nm. Images obtained from 6 randomly selected non-overlapping fields of culture dish were analyzed by ImageJ software [35].

### Statistical methods

Data are presented as mean ± standard deviation (SD) where applicable. Statistical analysis was performed using GraphPad Prism5.0 software and SPSS 16.0. Between-group differences were assessed using unpaired Student’s *t* test or one-way ANOVA, as appropriate. *P* values < 0.05 were considered indicative of statistical significance.

## Results

### MLKL and Cx43 were upregulated in the ipsilateral VPN after dMCAO

**Fig. 1:**
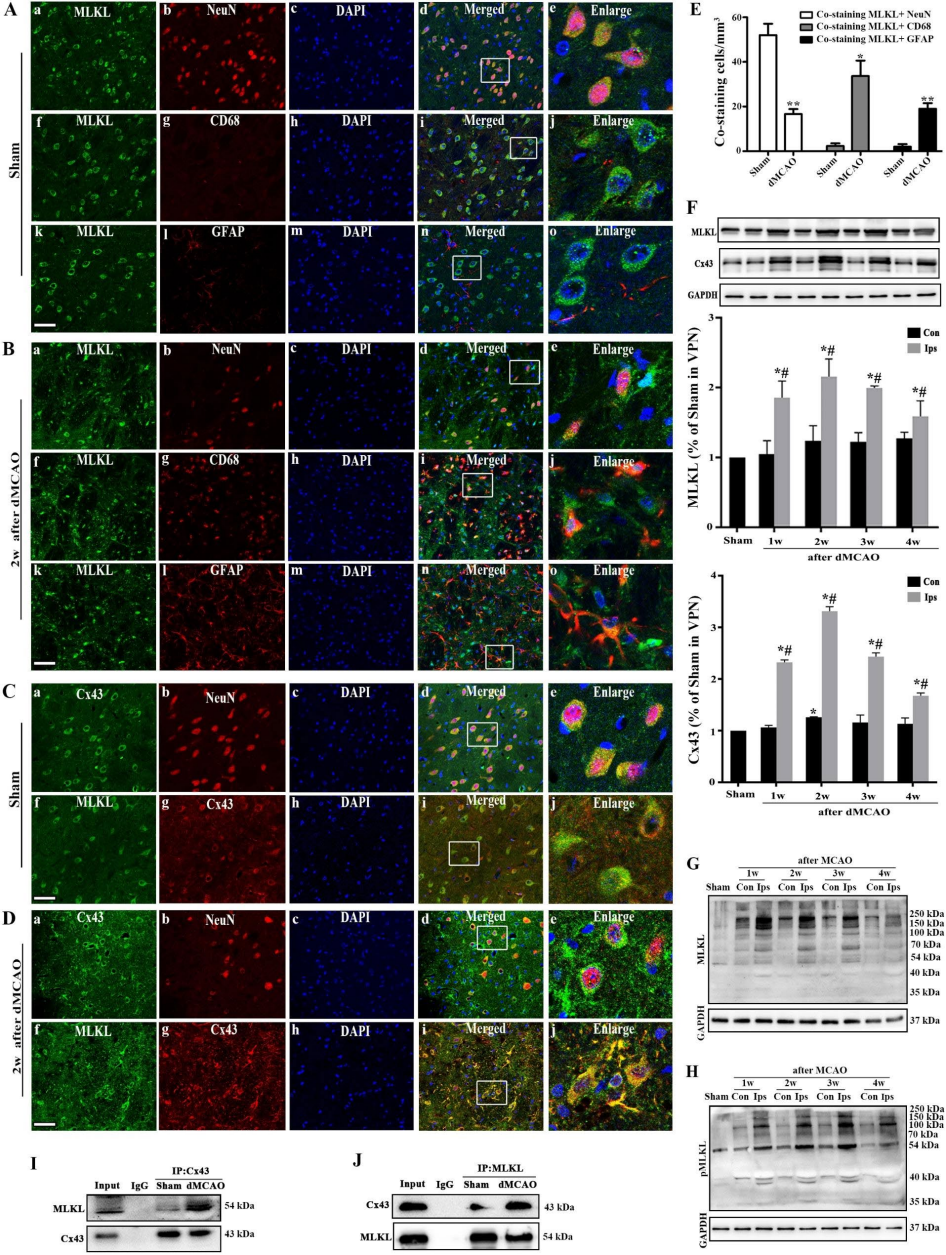
MLKL and Cx43 were upregulated in the ipsilateral VPN after dMCAO. (**A**, **B**) Representative microphotographs of the immunofluorescence assay of MLKL (green), NeuN/CD68/GFAP (red), and DAPI (blue) in VPN of Sham-operated group and 2w after dMCAO group, respectively (scale: 50 μm). (**C**, **D**) Representative microphotographs of the immunofluorescence assay of Cx43 and NeuN/MLKL in VPN of Sham-operated group and 2w after dMCAO group, respectively (scale: 50 μm). (**E**) Co-localization analysis of MLKL and NeuN, CD68 or GFAP. The histogram presents the quantitative analyses of co-staining of MLKL and NeuN, CD68, or GFAP (n = 6 in each group). (**F**) Representative immunoblots of MLKL and Cx43 expression, respectively, in VPN after dMCAO. The histogram presents the quantitative analyses of MLKL and Cx43 protein levels (n = 3 in each group). (**G**, **H**) Representative immunoblots of MLKL and pMLKL multimers expression, respectively, in VPN after dMCAO. (**I**, **J**) Co-immunoprecipitation analysis of MLKL and Cx43. Each bar represents mean ± standard deviation. ^*^P < 0.05 vs. Sham-operated group and ^#^*P* < 0.05 vs. contralateral to the lesion after dMCAO. dMCAO, distal middle cerebral artery occlusion model; MLKL, mixed lineage kinase domain-like protein; NeuN, neural nuclear antigen; GFAP, glial fibrillary acidic protein; CD68, cluster of differentiation 68; DAPI, 4′, 6-diamidine-2-phenylindole. Cx43, connexin 43; GAPDH, glyceraldehyde 3-phosphate dehydrogenase

To explore the involvement of necroptosis in the progression of secondary degeneration after dMCAO, we first detected MLKL level in VPN of rats by triple labeling with immunofluorescence assay. In the Sham-operated group, MLKL-positive labeling was found mainly in the neuron-like cells in the VPN (Fig. 1A). However, MLKL-positive labeling in the ipsilateral VPN was irregular, with diffuse staining in the cytoplasm, or as aggregates at 2 weeks after dMCAO. MLKL immunoreactivity was significantly greater in dMCAO compared with Sham groups; MLKL-positive labeling was observed not only in neurons but also in glial cells (Fig. 1B). Co-localization analysis showed that the predominant expression of MLKL was in the VPN neurons of Sham brains. After dMCAO, there was a decrease in the number of neurons in the ipsilateral VPN along with gliosis. MLKL-positive labeling in neurons was decreased compared with Sham groups, and the majority of MLKL-positive cells showed co-expression of CD68 or GFAP (Fig. 1E). In the Sham group, Cx43 was predominantly expressed in neurons (Fig. 1C a-e). However, following dMCAO, we observed clear co-localization of Cx43 expression in both neurons and glial-like cells in the VPN (Fig. 1D a-e). We also incorporated an analysis of CX43 and MLKL colocalization in VPN following dMCAO, we observed co-localization of Cx43 and MLKL in both neurons-like and glial-like cells in the VPN (Fig. 1C f-j, Fig. 1 Df-j). Consistent with the results of immunofluorescence analysis, Western blot analysis of MLKL and MLKL oligomers showed increased expression of the above factors in the ipsilateral VPN after dMCAO (Fig. 1F, G, H). Consistent with the results of MLKL, the expression of Cx43 was greatly increased in VPN of dMCAO rats at 2 weeks (Fig. 1F). Co-immunoprecipitation analysis confirmed the interaction of MLKL and Cx43. An obvious increase in the Cx43-MLKL interaction was observed at 2 weeks in the dMCAO group (Fig. 1I). The reverse co-IP was also proved the MLKL-Cx43 interaction (Fig. 1J).

### MLKL and Cx43 were upregulated after TSZ-mediated necroptosis in SH-SY5Y cells

**Fig. 2:**
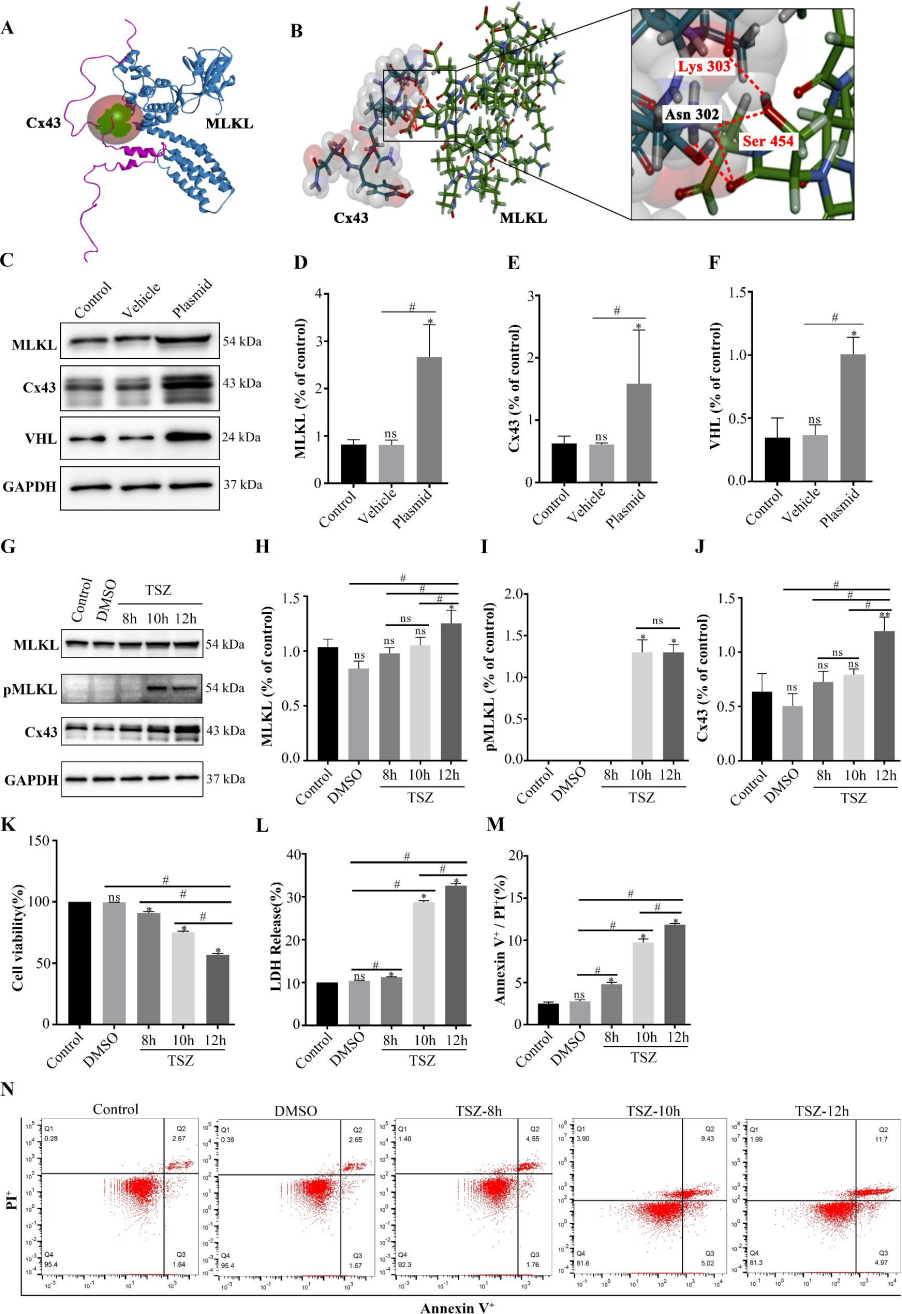
MLKL and Cx43 were upregulated after TSZ-mediated necroptosis in SH-SY5Y cells. (**A**, **B**) Molecular docking analysis of Cx43 and MLKL. (**C**). Western blots showing the protein expressions of MLKL, Cx43, and VHL in SH-SY5Y cells after transfection with plasmids. (**D**–**F**) Histogram showing the quantitative analyses of MLKL, Cx43, and VHL protein levels in SH-SY5Y cells after transfection with plasmids (n ≥ 3 in each group). (**G**) Western blots showing the protein expressions of Cx43, MLKL, and p-MLKL in SH-SY5Y cells after 8, 10, and 12 h of treatment with TSZ. (**H**–**J**) Histogram showing the quantitative analyses of MLKL, pMLKL, and Cx43 protein levels in SH-SY5Y cells after 8, 10, and 12 h of treatment with TSZ (n ≥ 3 in each group). (**K**) Viability of SH-SY5Y cells after 8, 10, and 12 h of treatment with TSZ (n ≥ 3 in each group). (**L**) Release of LDH in SH-SY5Y cells after 8, 10, and 12 h of treatment with TSZ (n ≥ 3 in each group). (**M**) Flow cytometry results: quantitative analysis of necroptotic cells after 8, 10, and 12 h of treatment with TSZ (n ≥ 3 in each group). (**N**) Representative photographs of flow cytometry analysis of necroptotic cells after 8, 10, and 12 h of treatment with TSZ. Each bar represents the mean ± standard deviation. ^*^P < 0.05 vs. control group and ^#^*P* < 0.05 vs. all groups except control group. MLKL, mixed lineage kinase domain-like protein; Cx43, connexin 43; VHL, von Hippel-Lindau; GAPDH, glyceraldehyde 3-phosphate dehydrogenase; LDH, lactate dehydrogenase; TSZ, TNFα + Smac mimetic + ZVAD-FMK; DMSO, dimethyl sulfoxide; PI, propidium iodide

Molecular docking analysis indicated a high binding affinity of Cx43 with MLKL (Bond dissociation energy =-78.43 kcal/ mol). As shown in Fig. 2A and B, MLKL Ser 454 interacted with Lys 303 and Asn 302 residues of Cx43 by forming a hydrogen bond (Fig. 2A, B). To further study neuronal necroptosis in vitro, we successfully transfected plasmids MLKL and Cx43 in SH-SY5Y neurons (Fig. 2C-F). The human neuroblastoma SH-SY5Y cells were subjected to TNFα-mediated necroptosis using conventional stimuli: TNFα (20 ng/mL), Smac mimetic (100 nM), and ZVAD-FMK (20 μM), abbreviated as TSZ. TNFα serves to activate TNFR1. The Smac mimetic inhibits cIAP-mediated ubiquitination of RIPK1. ZVAD-FMK functions as a the pan-caspase inhibitor [48]. After 10 and 12 h of TSZ treatment, p-MLKL increased significantly, the expressions of Cx43 and MLKL were up-regulated and significant after 12 h. (Fig. 2G-J). The CCK-8 assay was used to determine the viability of SH-SY5Y cells after treatment with TSZ for different time-periods. After 10 and 12 h of treatment with TSZ, the activity of SH-SY5Y cells showed a significant decrease (Fig. 2K). Previous studies have shown that LDH is an indicator of cellular necrosis [28, 49] and the double-labeled (PI+/Annexin V+) cells are considered as necroptotic cells [47]. As shown in Fig. 2L-N, LDH release and the percentage of PI+/Annexin V + cells (necroptotic) in the TSZ group were evidently increased at 10 and 12 h compared with the control group. Finally, we determined that the necroptosis induction regimen of SH-SY5Y cells was as follows: firstly, ZVAD-FMK (20 μM) and Smac mimetics (100 nM) were pretreated for 30 min, and then TNF-α (human) (20 ng/mL) was added for 10 h.

### Interaction between MLKL and Cx43 inhibits the K48-linked ubiquitination of Cx43 in necroptotic SH-SY5Y cells

**Fig. 3:**
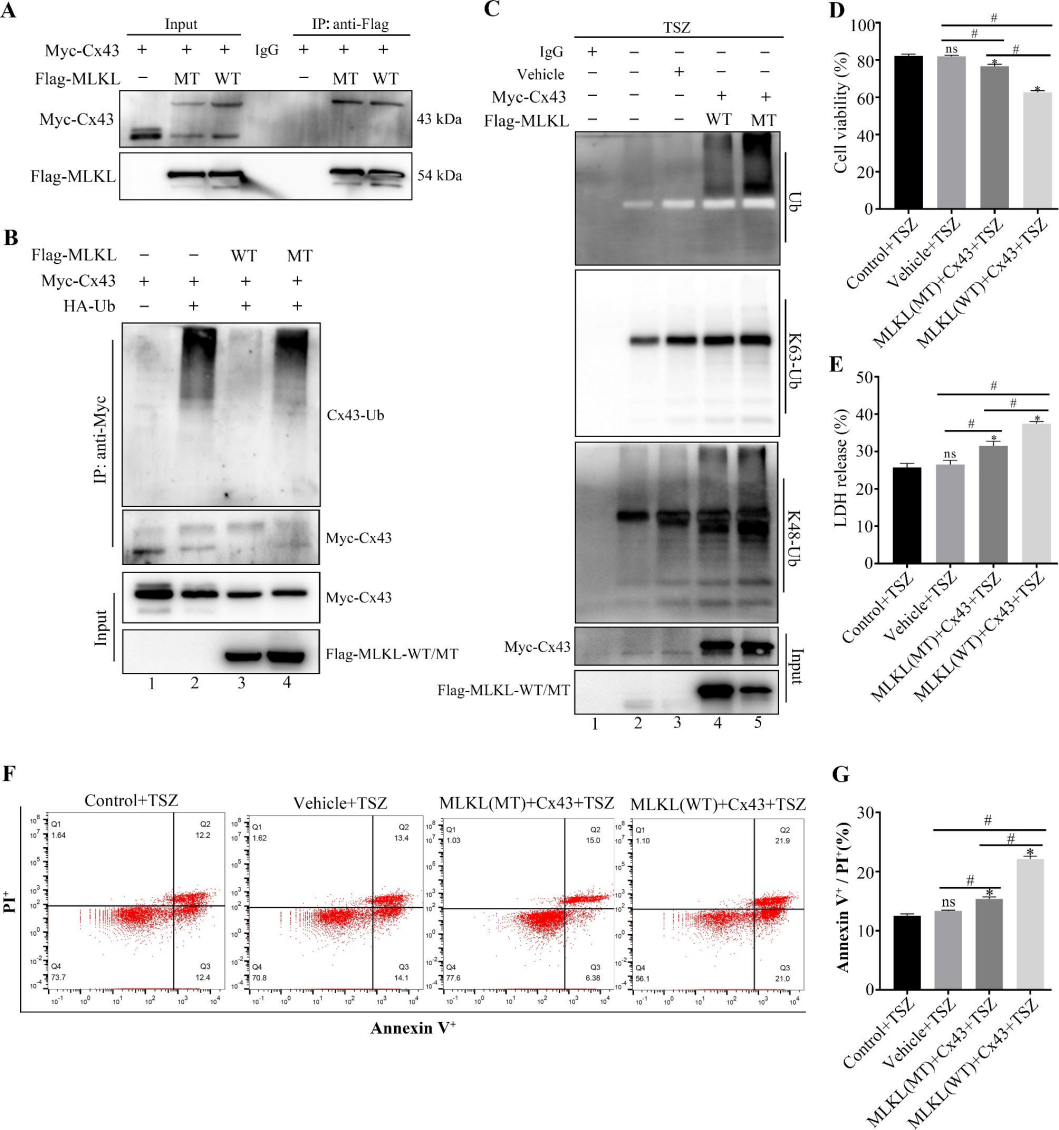
Interaction between MLKL and Cx43 inhibits the K48-linked ubiquitination of Cx43 in necroptotic SH-SY5Y cells. (**A**) Co-immunoprecipitation to detect the interaction between Cx43, MLKL(WT), and MLKL(S454A) in SH-SY5Y cells. (**B**) Co-immunoprecipitation and Western blot to detect Cx43 ubiquitination in SH-SY5Y cells post MLKL(WT) and MLKL(S454A) transfection. The ubiquitination level of Cx43 was significantly upregulated in Cx43 + Ub + MLKL(S454A) transfection group (lane 4) in contrast to Cx43 + Ub + MLKL(WT) transfection group (lane 3). (**C**) Co-immunoprecipitation and Western blot to detect K48-linked ubiquitination of Cx43 in SH-SY5Y cells post MLKL(WT) and MLKL(S454A) transfection. The K48-linked ubiquitination level of Cx43 was significantly upregulated in Cx43 + MLKL(S454A) transfection group (lane 5) in contrast to Cx43 + MLKL(WT) transfection group (lane 4). (**D**) Viability of SH-SY5Y cells after transfection with MLKL(WT), MLKL(S454A), and Cx43(WT) plasmids (n ≥ 3 in each group). (**E**) Release of LDH in SH-SY5Y cells after transfection with MLKL(WT), MLKL(S454A), and Cx43(WT) plasmids (n ≥ 3 in each group). (**F**) After transfecting SH-SY5Y cells with MLKL(WT), MLKL(S454A), and Cx43(WT) plasmids, representative photographs of flow cytometry analysis of necroptotic cells. (**G**) After transfecting SH-SY5Y cells with MLKL(WT), MLKL(S454A). and Cx43(WT) plasmids, quantitative analysis of necroptotic cells by flow cytometry analysis (n ≥ 3 in each group). Each bar represents mean ± standard deviation. ^*^P < 0.05 vs. control group and ^#^*P* < 0.05 vs. all groups except the control group. MLKL, mixed lineage kinase domain-like protein; Cx43, connexin 43; WT, wild type; MT, mutant; Ub, ubiquitin; TSZ, TNFα + Smac mimetic + ZVAD-FMK; PI, propidium iodide

Molecular docking analysis confirmed that Cx43 carboxyl terminal Asn 302 and Lys303 interacted with Ser454 of MLKL through hydrogen bond (Fig. 2A, B). To further clarify the mechanism of interaction between Cx43 and MLKL, we first transfected SH-SY5Y cells with MLKL(WT), MLKL(S454A) and Cx43(WT) plasmids. Co-immunoprecipitation analysis revealed a direct interaction between MLKL(WT), MLKL(S454A), and Cx43 (Fig. 3A). Cx43 immunoblots of ubiquitin Co-IP analysis demonstrated that Cx43 was ubiquitylated in the presence of Cx43 and ubiquitin (lane 2), in contrast to non-ubiquitylated Cx43 principally found in lane 1. Addition of MLKL(WT) significantly decreased the ubiquitination of Cx43 (lane 3); this observation suggests that the ubiquitylates of Cx43 were possibly regulated by MLKL. Our experiments thus far suggested a potential interaction of Cx43 with Ser454 of MLKL; we speculated that disrupting the interaction between Cx43 and MLKL may affect the ubiquitination level of Cx43. After transfection of SH-SY5Y cells with the MLKL mutant (S454A), we observed a significant upregulation of the ubiquitination level of Cx43, which further confirmed this view (Fig. 3B lane 4). After treatment with TSZ for 10 h, S454A mutant of MLKL increased the K48-linked ubiquitination of Cx43 (Fig. 3C, lane 5, K48-Ub) in contrast to MLKL (WT) (lane 4), but not K63-linked ubiquitination (Fig. 3C, lane 5, K63-Ub). These findings suggest that MLKL inhibits Cx43 ubiquitination, mainly by inhibiting K48-linked ubiquitination of Cx43. S454A mutant of MLKL inhibited TSZ-induced cell death (Fig. 3D) and LDH release in necroptotic cells (Fig. 3E). Flow cytometry analysis further confirmed that the S454A mutant of MLKL decreased the percentage of PI^+^/Annexin V^+^ necroptotic cells (Fig. 3F, G). These results suggest that MLKL selectively promotes the K48-linked ubiquitination of Cx43 in necroptosis.

### VHL is an E3 ubiquitin ligase for Cx43, and MLKL competes with VHL for binding to Cx43

**Fig. 4:**
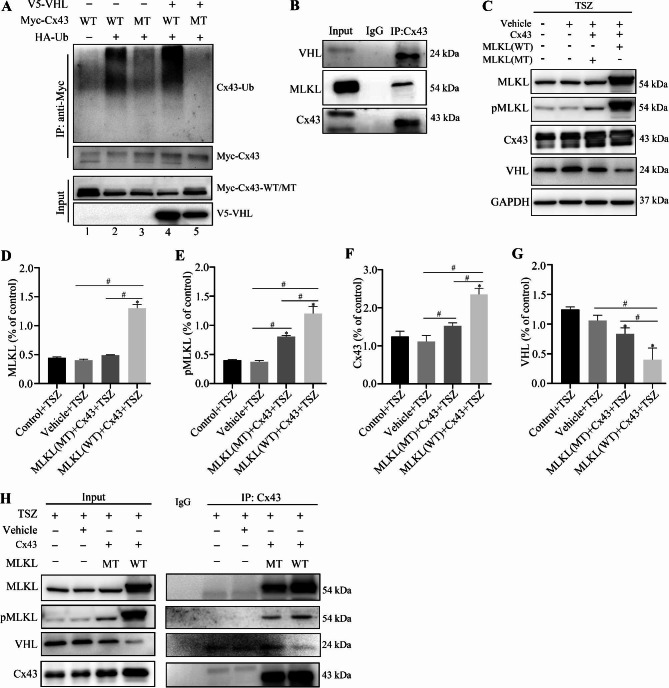
VHL is an E3 ubiquitin ligase for Cx43, and MLKL competes with VHL for binding to Cx43. (**A**) Co-immunoprecipitation and Western blot to detect Cx43 ubiquitination in SH-SY5Y cells post transfection with Cx43(WT), Cx43(K303G), Ub, and VHL. The ubiquitination level of Cx43 was significantly decreased in VHL + Ub + Cx43 (K303G) transfection group (lane 5) in contrast to VHL + Ub + Cx43 (WT) transfection group (lane 4). (**B**) Co-immunoprecipitation and Western blot to detect the interaction among Cx43, MLKL, and VHL in SH-SY5Y cells. (**C**) Representative immunoblots of MLKL, pMLKL, Cx43, and VHL expression in SH-SY5Y cells post TSZ treatment, Cx43, MLKL(WT), and MLKL(S454A) transfection. **D**, **E**, **F**, **G**) Histogram showing the quantitative analyses of MLKL, pMLKL, Cx43, and VHL protein levels in SH-SY5Y cells after TSZ treatment and plasmids transfection (n ≥ 3 in each group). **H**) Co-immunoprecipitation and Western blot to detect the interaction among MLKL, pMLKL, VHL, and Cx43 in SH-SY5Y cells after TSZ treatment and plasmids transfection. Each bar represents mean ± standard deviation. ^*^P < 0.05 vs. control group and ^#^*P* < 0.05 vs. all groups except the control group. MLKL, mixed lineage kinase domain-like protein; Cx43, connexin 43; WT, wild type; MT, mutant; Ub, ubiquitin; VHL, von Hippel-Lindau; GAPDH, glyceraldehyde 3-phosphate dehydrogenase; TSZ, TNFα + Smac mimetic + ZVAD-FMK

To further explore the potential factors affecting the ubiquitination and degradation of Cx43 in neurons, we used UbiBrowser software (http://ubibrowser.ncpsb.org) to predict the E3 ligase of Cx43 ubiquitin [50]. The prediction results showed that Von Hippel-Lindau (VHL) may be one of the E3 ubiquitin ligases that regulated Cx43 ubiquitination at Lys 303. Coincidentally, molecular docking results showed that Cx43 Lys303 interacted with Ser454 of MLKL through H-bond formation (Fig. 2A, B). To further clarify whether VHL can regulate Cx43 ubiquitination at Lys 303, we first transfected SH-SY5Y cells with VHL, Cx43(WT), Cx43(K303G), and Ub plasmids. Co-IP analysis confirmed that VHL increased the ubiquitination of Cx43 in VHL + Ub + Cx43(WT) transfection group (Fig. 4A, lane 4). However, after mutating the Cx43 Lys303 (K303G), the ubiquitination level of Cx43 was significantly downregulated (Fig. 4A, lane 5). Co-IP assay revealed a direct interaction between VHL, MLKL, and Cx43 (Fig. 4B). After treatment with TSZ for 10 h, compared with control groups, the expression levels of MLKL, pMLKL, and Cx43 were largely increased while the expression level of VHL decreased (Fig. 4C-G). However, after mutating the MLKL Ser454 (S454A), the interaction of VHL with Cx43 and MLKL decreased (Fig. 4H). This possibly indicates that the mutant MLKL binds less to Cx43, allowing for more VHL to bind to the Cx43 K303 site. These results suggest that MLKL may compete with VHL for binding to Cx43.

### Interaction of MLKL Ser454 with Cx43 can trigger the opening of Cx43 hemichannels, increasing intracellular Ca^2+^ and cell necroptosis

**Fig. 5:**
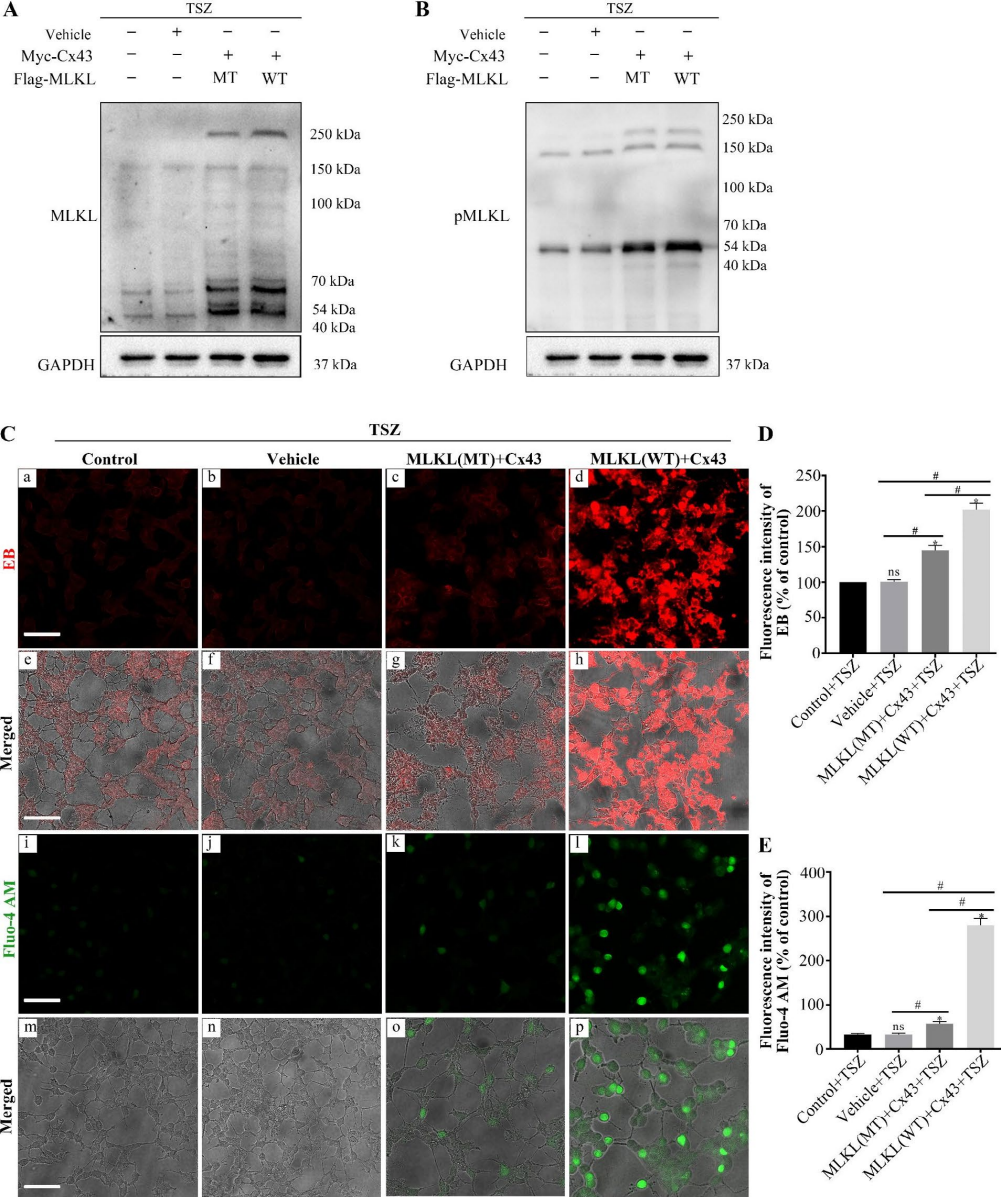
Interaction of MLKL Ser454 with Cx43 can trigger the opening of Cx43 hemichannels, leading to increased intracellular Ca^2+^, and cell necroptosis. **A**, **B**) Western blots to detect MLKL and pMLKL polymers in SH-SY5Y cells post TSZ treatment, Cx43, MLKL(WT), and MLKL(S454A) transfection. C (**a**-**h**) Dye uptake assay to detect Cx43 hemichannels in SH-SY5Y cells post TSZ treatment, Cx43, MLKL(WT), and MLKL(S454A) transfection. C (**i**-**p**) Fluo-4 AM to detect cytoplasmic Ca^2+^ concentration in SH-SY5Y cells post TSZ treatment, Cx43, MLKL(WT), and MLKL(S454A) transfection (scale: 50 μm). **D**) The histogram presents the quantitative analyses of EB fluorescence intensity (n = 6 in each group). **E**) The histogram presents the quantitative analyses of Fluo-4 AM fluorescence intensity (n = 6 in each group). MLKL, mixed lineage kinase domain-like protein; Cx43, connexin 43; WT, wild type; MT, mutant; GAPDH, glyceraldehyde 3-phosphate dehydrogenase; TSZ, TNFα + Smac mimetic + ZVAD-FMK; EB, ethidium bromide

We examined the effect of TSZ on MLKL polymer formation. As shown in Fig. 5A-B, we observed MLKL and pMLKL polymer formation after co-transfection of Cx43 and MLKL (WT). After mutating the MLKL Ser454 (S454A), there was a decrease in MLKL and pMLKL polymer formation. Further, to confirm whether MLKL-induced necroptosis exacerbates Cx43 hemichannel opening, we used EB to detect the hemichannel activity, since Ca^2+^ influx into cells acts as a downstream factor of MLKL during necroptosis [33]. Intracellular free calcium concentration was measured using Fluo-4 AM fluorescence indicator. Immunofluorescence assay showed that the co-transfection of Cx43 and MLKL (WT) can trigger the opening of Cx43 hemichannels, and intracellular Ca^2+^ increased after TSZ-induced necroptosis of SH-SY5Y cells. After mutating the MLKL Ser454 (S454A), the opened Cx43 hemichannels and intracellular Ca^2+^ decreased (Fig. 5C). Marked increases in EB and Fluo-4 AM fluorescence intensity were observed after co-transfection of Cx43 and MLKL (WT). In contrast, the treatment with mutating the MLKL Ser454 (S454A) attenuated MLKL-induced Cx43 hemichannel opening and Ca^2+^ influx (Fig. 5D, E).

## Discussion

**Fig. 6:**
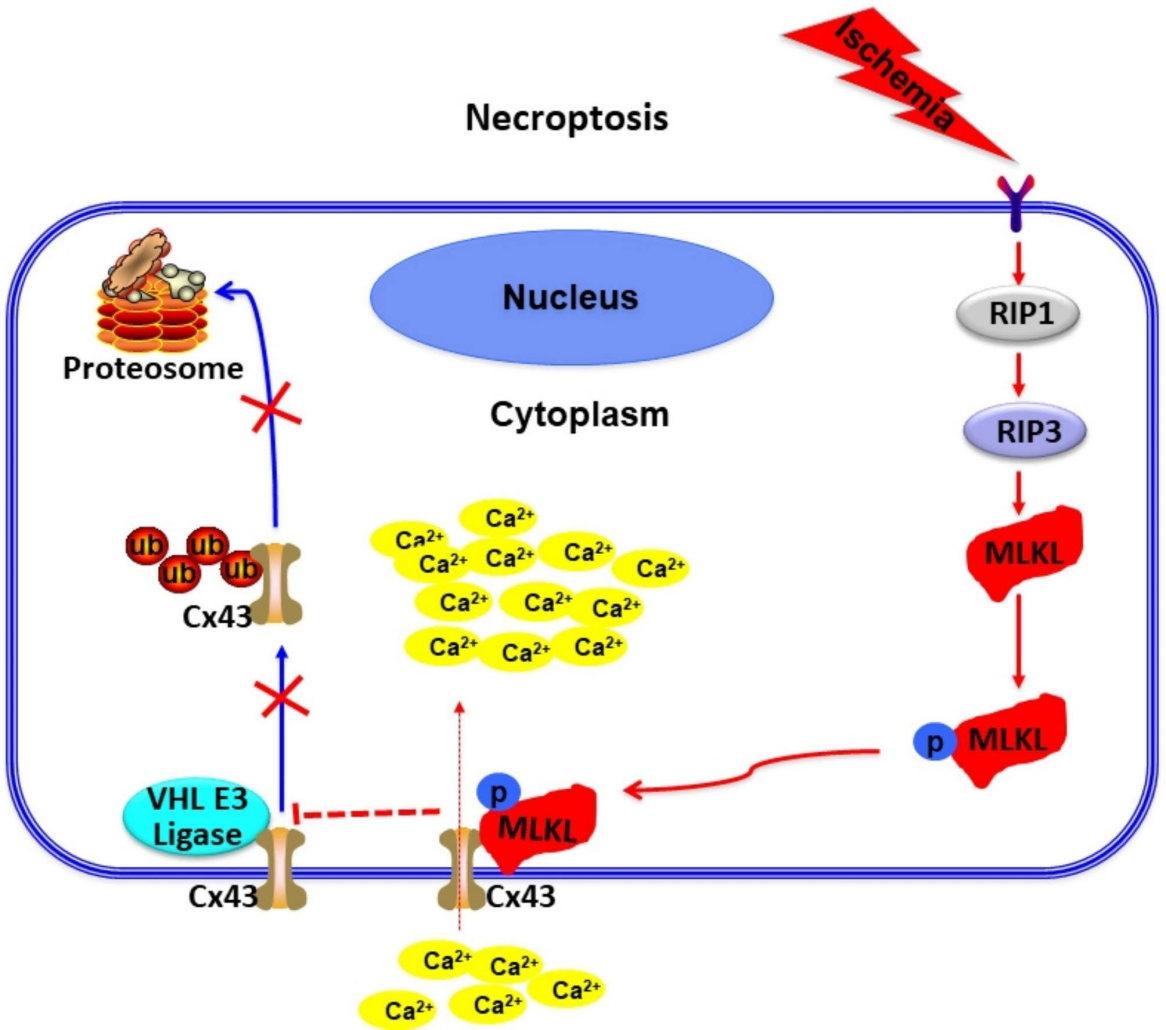
Schematic illustration of the mechanism by which MLKL regulates Cx43 ubiquitination and degradation to mediate necroptosis in ipsilateral thalamic neurons after focal cerebral infarction. The RIP1-RIP3-MLKL pathway was activated in the VPN of ipsilateral thalamus after focal cerebral ischemia. MLKL was up-regulated and translocated to the plasma membrane and then interacted with Cx43 at Lys 303, thereby inhibiting the ubiquitination of Cx43 at Lys 303 by VHL and the ubiquitin-proteasomal degradation of Cx43, promoting opening of Cx43 hemichannels, overloading intracellular calcium, and eventually leading to neuronal necroptosis in VPN

Secondary neurodegeneration in the thalamus after focal cerebral infarction is associated with poor psychosocial outcomes and quality of life of patients. Ischemic brain injury leads to neuronal cell death. Various studies have demonstrated the involvement of necroptotic cell death in the pathogenesis of cerebral infarction [51]. Treatment with specific inhibitor of necroptosis (Nec-1) was shown to prevent neuronal necroptosis by reducing the activation of RIP1/RIP3/MLKL and inhibiting its downstream signaling pathways [26]. However, the role of necroptosis in post-cerebral infarction secondary degeneration is not well characterized. In the current study, we have demonstrated for the first time that the executioner of necroptosis, MLKL was upregulated and formed multimers in the ipsilateral VPN after dMCAO. MLKL interacted with Cx43 at Lys 303, thereby inhibiting the ubiquitination of Cx43 at Lys 303 by VHL and the ubiquitin-proteasomal degradation of Cx43, promoting opening of Cx43 hemichannels, overloading intracellular calcium, and eventually leading to neuronal necroptosis in VPN (Fig. 6). To the best of our knowledge, this is the first study to provide comprehensive evidence of the involvement of Cx43 in cerebral ischemia-induced neuronal necroptosis mediated by a MLKL-dependent mechanism.

Necroptosis is a potential novel therapeutic target to prevent secondary neurodegeneration after ischemic stroke and, reduce the long-term sequelae of cerebral infarction. MLKL is currently believed to be the executioner in necroptosis. RIP3 phosphorylates MLKL, triggering MLKL oligomerization and its translocation to membrane leading to membrane disruption [25, 52]. To determine the involvement of MLKL in dMCAO-induced necroptosis of thalamic neurons, we examined the expression of MLKL. We found greatly increased expression of MLKL and MLKL oligomers in VPN of dMCAO rats at 2 weeks. Similarly, we observed the morphological changes of MLKL by immunofluorescence and co-localization analysis, and found neuron-dominant expression in the Sham operated group. However, at 2 weeks after dMCAO, MLKL-positive labeling was observed not only in neurons but also in glial cells (Fig. 1). A recent study has shown that cerebral ischemia can induce a rapid MLKL-mediated neuron-dominated necroptosis. After focal cortical ischemia in mice, necroptosis was observed in neurons at the early stage but in glial cells at the later stage [53]. Much research suggests that debris of dead neurons may trigger glia-mediated neuroinflammation, thus increasing neuronal death in neurodegenerative diseases [54]. Therefore, we speculate that this time-dependent transition of MLKL from neurons to glial cells reflects the pathological changes of cerebral ischemia and may lead to the development of secondary degeneration in thalamus.

Although the core necroptotic pathway has been well established, the details of the mechanism by which MLKL translocates to the membrane and causes membrane rupture remains an open area of research. Recent evidence suggests that membrane translocation of MLKL is necessary but not sufficient to induce cell death [29], and that there are several other potential downstream proteins or pathways that mediate necroptosis [34, 55]. During necroptosis, an influx of Ca^2+^ has been described, although its role in necroptosis has been contested [25, 33]. Gong et al. demonstrated that an influx of Ca^2+^ occurred following activation of MLKL, prior to the exposure of phosphatidylserine, and was dependent on the presence of MLKL [56]. Based on the above evidence, we speculated the potential involvement of other Ca^2+^-permeable channels downstream of MLKL.

Cx43 is one of the key proteins involved in inter-cellular ion exchange. Under pathological conditions such as ischemia and hypoxia, a large number of Cx43 hemichannels are opened, thereby increasing the intracellular Ca^2+^ concentration [57]. We further sought to confirm whether Cx43 is a downstream protein of MLKL that mediates intracellular calcium overload. Our experiments revealed the interaction of MLKL Ser 454 with Lys303 and Asn302 residues of Cx43 via formation of a hydrogen bond. In the SH-SY5Y cell necroptosis model, we confirmed the interaction between Cx43 and the Ser454 residue of MLKL by overexpression and site-directed mutagenesis. Ubiquitin is known to regulate the functions of Cx43. Previous study has confirmed that Cx43 K63-linked ubiquitylation on lysines 264 and 303 regulates gap junction internalization [43]. K48-linked ubiquitination targets substrates for degradation by proteasomes, whereas K63-linked ubiquitination modulates protein activation or signal transmission [58]. The present study is the first to provide evidence that MLKL competed with VHL to inhibit Cx43 ubiquitination (Fig. 4), mainly inhibiting K48-linked ubiquitination of Cx43 (Fig. 3). Therefore, we speculated that when cells underwent necroptosis, the ubiquitination and degradation of Cx43 on the cell membrane decreased, the number of Cx43 hemichannels which allowed passage of Ca^2+^ to increase, and the intracellular Ca^2+^ concentration rised. After TSZ-induced necroptosis, wild-type MLKL could profusely open Cx43 hemichannels and cause intracellular calcium overload. However, mutation of the Ser454 residue of MLKL reduced the opening of hemichannels and calcium influx, thus reducing neuronal necroptosis (Fig. 5). This finding confirms that the interaction between Cx43 and MLKL is crucial for triggering neuronal necroptosis in thalamic degeneration secondary to cerebral ischemia. Further in vivo mechanistic studies are required to validate the therapeutic potential of MLKL in secondary neurodegeneration after ischemic stroke.

In conclusion, we provide comprehensive evidence of the involvement of that necroptosis in the pathogenesis and development of secondary neurodegeneration after ischemic stroke. Our results indicated that MLKL regulates Cx43 ubiquitination and degradation and participates in neuronal necroptosis. With the development of detection techniques and methods, more neuronal death patterns in secondary neurodegeneration after cerebral infarction may be discovered in the future. This study lays a foundation for future research of necroptosis in secondary neurodegeneration after ischemic stroke, and provides meaningful insights for the development of therapeutic straties.

## Conclusion

As shown in Fig. 6, in the present study, we have demonstrated that the injury in ipsilateral thalamus after focal cortical infarction was regulated by Cx43 via a MLKL-dependent mechanism. These findings indicate that the interaction between Cx43 and MLKL is critical for neuronal necroptosis in thalamic degeneration secondary to cerebral ischemia. And these results provide promising therapeutic targets for the ischemic strokes.

## Data Availability

Data supporting the findings of this study are available upon a reasonable request to the corresponding author.
